# Feasibility of using ring‐mounted Halcyon Linac for single‐isocenter/two‐lesion lung stereotactic body radiation therapy

**DOI:** 10.1002/acm2.13555

**Published:** 2022-02-07

**Authors:** Damodar Pokhrel, Aaron Webster, Richard Mallory, Justin Visak, Mark E. Bernard, Ronald C. McGarry, Mahesh Kudrimoti

**Affiliations:** ^1^ Medical Physics Graduate Program Department of Radiation Medicine University of Kentucky Lexington Kentucky USA

**Keywords:** co/non‐coplanar, Halcyon RDS, lung SBRT, single‐isocenter, treatment efficiency, VMAT

## Abstract

**Purpose:**

To demonstrate the plan quality and delivery efficiency of volumetric‐modulated arc therapy (VMAT) with the Halcyon Linac ring delivery system (RDS) in the treatment of single‐isocenter/two‐lesion lung stereotactic body radiation therapy (SBRT).

**Materials/methods:**

Sixteen previously treated non‐coplanar VMAT single‐isocenter/two‐lesion lung SBRT plans delivered with SBRT‐dedicated C‐arm TrueBeam Linac were selected. Prescribed dose was 50 Gy to each lesion over five fractions with treatment delivery every other day and AcurosXB algorithm as the final dose calculation algorithm. TrueBeam single‐isocenter plans were reoptimized for Halcyon Linac with coplanar geometry. Both TrueBeam and Halcyon plans were normalized for identical combined target coverage and evaluated. Conformity indices (CIs), heterogeneity index (HI), gradient index (GI), gradient distance (GD), and *D*_2cm_ were compared. The normal lung V5Gy, V10Gy, V20Gy, mean lung dose (MLD), and dose to organs at risk (OAR) were evaluated. Treatment delivery parameters, including beam‐on time, were recorded.

**Results:**

Halcyon plans were statistically similar to clinically delivered TrueBeam plans. No statistical differences in target conformity, dose heterogeneity, or intermediate‐dose spillage were observed (all, *p* > 0.05). Halcyon plans, on average, demonstrated statistically insignificant reduced maximum dose to most adjacent OAR and normal lung. However, Halcyon yielded statistically significant lower maximal dose to the ribs (*p* = 0.041) and heart (*p* = 0.026), dose to 1 cc of ribs (*p* = 0.035) and dose to 5 cc of esophagus (*p* = 0.043). Plan complexity slightly increased as seen in the average increase of total monitor units, modulation factor, and beam‐on time by 480, 0.48, and 2.78 min, respectively. However, the estimated overall treatment time was reduced by 2.22 min, on average. Mean dose delivery accuracy of clinical TrueBeam plans and the corresponding Halcyon plans was 98.9 ± 0.85% (range: 98.1%–100%) and 98.45 ± 0.99% (range: 97.9%–100%), respectively, demonstrating similar treatment delivery accuracy.

**Conclusion:**

SBRT treatment of synchronous lung lesions via single‐isocenter VMAT on Halcyon RDS is feasible and dosimetrically equivalent to clinically delivered TrueBeam plans. Halcyon provides excellent plan quality and shorter overall treatment time that may improve patient compliance, reduce intrafraction movement, improve clinic efficiency, and potentially offering lung SBRT treatments for underserved patients on a Halcyon only clinic.

## 1 INTRODUCTION

Lung cancer is the leading cause of cancer‐related mortality in North America with an increasing number of patients presenting with stage I/II non‐small‐cell lung cancer (NSCLC).^1^ In many cases, these pulmonary cancer patients present with comorbidities that preclude surgical resection.^2^, ^3^ For these patients, stereotactic body radiation therapy (SBRT) is the standard‐of‐care due to its extremely effective 3‐year local control rates of up to 97% in the treatment of solitary lung lesions when compared to conventional lung radiotherapy.^4^ SBRT is well‐tolerated compared to surgery due to its low rates of treatment induced toxicity.^5^, ^6^, ^7^, ^8^ Additionally, for the large and growing cohort presenting with multiple primary lung tumors or oligometastatic disease, several studies have shown SBRT to be a safe and effective treatment, including phase I/II trials that yielded 1 and 2‐year local control rates of 100% and 96%, respectively.^9^, ^10^, ^11^ The effectiveness of lung SBRT depends heavily on the employed delivery technique and its ability to provide increased target conformity and rapid dose fall while accounting for tumor motion due to respiration.^12^, ^13^ Successful management of these challenging patient cases requires rapid high‐dose fall‐off in order to spare adjacent dose limiting organs and normal lung tissue. This can be especially difficult for pulmonary distressed patients with multiple lung lesions as SBRT approaches frequently require long treatment times that can reduce patient compliance and increase intrafraction motion.^14^, ^15^, ^16^

Stereotactic treatment of multiple lung lesions can be delivered asynchronously by using a multi‐isocentric approach, often at the cost of significant increases in treatment planning, patient set up and treatment delivery time. Alternatively, a single‐isocenter technique can be utilized to treat multiple lung lesions synchronously. While the latter method can provide substantially shorter treatments, the effectiveness relies primarily on the accuracy of patient set up and the precision of treatment delivery accentuates errors based on various geometric factors.^17^ Recently, Varian RapidArc® planning with volume‐modulated arc therapy (VMAT) (Varian Medical Systems, Palo Alto, CA, USA) has emerged as an effective treatment planning and delivery technique in lung SBRT. This advanced treatment offers higher precision treatments with steep dose gradients, increased sparing of organs at risk (OAR) and faster delivery times when compared to previous techniques.^18^, ^19^ Introduction of flattening‐filter‐free (FFF) beams in VMAT has further enhanced these capabilities with higher dose rates, less out of target dose, and reduced head contamination including improving target dose coverage at lung–tissue interface compared to flattened beams.^20^ Moreover, these benefits may be enhanced for the synchronous treatment of multiple lung lesions when coupled with an advanced Linac design.

Recently, Varian Medical Systems introduced a new jawless, single energy, ring‐mounted delivery system, the Halcyon (V2.0) medical linear accelerator (ring delivery system, RDS).^21^ The novel system offers a gantry rotation speed up to 4 revolution/min and is equipped with a 6MV‐FFF beam with a maximum output rate of 800 monitor units (MU)/min. The Halcyon output rate is substantially lower than that of the 6MV‐FFF beam on C‐arm TrueBeam Linac of 1400 MU/min. Halcyon Linac's mean energy and nominal depth of maximum dose is 1.3 MeV and 1.3 cm, whereas the TrueBeam Linac's 6MV‐FFF yields 1.4 MeV and 1.5 cm, respectively. In contrast to TrueBeam's single layer Millennium 120 MLC design, Halcyon RDS is equipped with a dual‐layer stacked and staggered MLC design (SX2) that, like the Millennium 120, offers an effective resolution of 5 mm MLC width at the treatment isocenter. Halcyon has a field size restriction of 28 × 28 cm^2^, but due to the novel MLC design, can achieve full leaf interdigitation with a maximal leaf travel of 28 cm. Halcyon also boasts a reduced beam penumbra as seen in the smaller dosimetric leaf gap of 0.1 mm due to its improved focused tip design. Moreover, the SX2 has a faster leaf acceleration and velocity (200 cm/s/s and up to 5 cm/s, respectively) at the plane of isocenter compared to the Millennium 120 MLC (50 cm/s/s and 2.5 cm/s, respectively). The unique stacked design allows for ultra‐low leakage and transmission of roughly 0.4% which may improve treatment accuracy.^22^, ^23^ Additionally, Halcyon comes equipped with fast on‐board imaging capable of up to 15‐s kilovoltage cone beam computed tomography (kV‐CBCT) imaging with an advanced iterative CBCT (iCBCT) reconstruction algorithm.^24^ Halcyon also reduces patient set up time significantly with the convenience of a fully automated one‐step patient set up approach where isocenter shifts are automatically applied.^21^

In the stereotactic treatment regime, there have been limited studies that demonstrate the feasibility of using Halcyon RDS for the treatment of both intracranial and extracranial disease.^25^, ^26^, ^27^, ^28^, ^29^ In a planning study comparing TrueBeam and Halcyon, Petroccia et al.^27^ concluded that clinically acceptable spine SBRT is possible with Halcyon RDS. In the clinical setting, Pokhrel et al.^28^, ^30^ reported successful clinical implementation of Halcyon in the delivery of prostate and single lung lesion SBRT treatments. In the single‐isocenter/multi‐lesion setting, Li et al.^29^ conducted a retrospective planning study that demonstrated Halcyon 2.0 to be capable of generating similar plan quality to C‐arm TrueBeam in the treatment of six to 10 brain lesions with a single‐isocenter VMAT approach. Although these studies show promise for increasing Halcyon's role in the SBRT treatment delivery, there is currently no literature addressing the feasibility of using Halcyon RDS in the treatment of multiple lung lesions with SBRT. In this study, we conduct a retrospective evaluation of plan quality and delivery efficiency using a ring‐mounted Halcyon Linac in the delivery of single‐isocenter/two‐lesion VMAT lung SBRT for 16 patients previously treated on a TrueBeam Linac. We also assess clinical acceptability by evaluating adherence to RTOG‐0813/NRG‐BR001 lung SBRT protocol requirements.^3^, ^11^

## 2 MATERIALS AND METHODS

Upon attaining Institutional Review Board approval, a total of 16 patients with stage I–II NSCLC metastatic lesions previously treated to 50 Gy in five fractions with single‐isocenter/two‐lesion lung SBRT on a TrueBeam Linac were selected for this retrospective study.

### 2.1 Patient set up and target delineation

Patient immobilization was achieved using the Body Pro‐Lok™ platform (CIVCO system, Orange City, IA, USA). All patients were placed in the supine position with their arms over their head and if possible, diaphragmatic compression was utilized to reduce motion to no more than 1.0 cm. Simulation was conducted using a free‐breathing 3D‐CT scan with GE Lightspeed 16 slice CT scanner (General Electric Medical Systems, Waukesha, WI, USA) in helical mode. Gross target volumes (GTV) were delineated based on observable tumor mass followed by creation of planning target volumes (PTV) via 1.0 cm expansion of the GTV in the superior–inferior direction and a 0.5 cm expansion laterally.^3^ For patients not able to tolerate abdominal compression, in addition to free‐breathing 3D‐CT, 4D‐CT scans were obtained using the Varian RPM system (version 1.7). Maximum intensity projection (MIP) images were then created and co‐registered to free‐breathing 3D‐CT scans to facilitate internal target volume (ITV) delineation. PTV were then defined by a 0.5 cm expansion of the ITV. All treatment planning was performed on the free‐breathing 3D‐CT dataset. As per the requirements established in RTOG‐0813, all relevant critical OARs were delineated to include spinal cord, heart/pericardium, bronchus, trachea, esophagus, skin, ribs (right, left, and combined), and normal uninvolved lung (right, left, combined). Table 1 summarizes the lesion characteristics and geometries for all 16 lung SBRT patients used in this study.

**TABLE 1:** Main tumor characteristics of the 16 lung stereotactic body radiation therapy (SBRT) patients included in this study

Parameters	Mean ± SD (range or *n*: no. of patients)
Tumor 1, PTV1 (cc)	25.9 ± 23.4 (4.9–81.2)
Tumor 2, PTV2 (cc)	20.8 ± 12.9 (6.4–41.0)
Tumor location (left/right/bilateral lungs)	*n* = 5/3/8
Distance to isocenter (cm)	5.5 ± 2.5 (2.4−10.2)
Normal lung volume (cc)	3526.0 ± 1180.0 (1892.0−6542.0)

*Note*: Each patient had two tumors. Dose was 50 Gy in five fractions to each tumor.

Abbreviations: PTV, planning target volume; SD, standard deviation.

### 2.2 Treatment planning

#### 2.2.1 Clinical TrueBeam VMAT plans

All patients were treated with a highly conformal VMAT plan using two to four non‐coplanar (±5°–10° couch rotations) or coplanar partial/full arcs on a TrueBeam Linac equipped with a Millennium 120 MLC using the 6MV‐FFF beam with a maximum output rate setting of 1400 MU/min. Due to clearance issue, four patient's plans consisted entirely of coplanar beam geometry. The TrueBeam couch top and the SBRT board were inserted as planning structures. A single‐isocenter was placed approximately equidistant between the two lesions. As the isocenter does not always need to be precisely between the lesions, an offset was applied to facilitate gantry rotation for the partial arcs. Collimator angles for these arcs were chosen to minimize tongue and groove multileaf collimators (MLC) leakage and dose bridging throughout gantry rotation. Jaw tracking option was also used to reduce leakage dose outside the field. The isocenter to tumor distance was the 3D linear distance from the placement of the single isocenter to the geometric center of each lesion. A dose of 50 Gy in five fractions was prescribed to the 70%–80% isodose line and normalized such that 95% of each PTV received 100% of the prescription dose with a target maximum GTV dose of 120%–130%. Clinical plans were generated using the Eclipse TPS inverse optimizer (Photon Optimizer, PO version 13.6 or 15.6) with final dose calculation using the AcurosXB algorithm with 1.25 mm dose calculation grid size (CGS), tissue heterogeneity corrections,^31^, ^32^ and dose to medium reporting mode enabled. Planning objectives were established using NRG‐BR001 or RTOG‐0813 guidelines.^3^, ^11^ Patients were treated every other day per lung SBRT protocol, and an online pre‐treatment CBCT scan was performed prior to each treatment for patient set up corrections.

#### 2.2.2 Halcyon VMAT plans

For comparison, all TrueBeam single‐isocenter plans were reoptimized for Halcyon RDS coplanar geometry with two to four partial/full arcs using 6MV‐FFF beam and a maximum output rate of 800 MU/min. The TrueBeam couch structure was replaced by the Halcyon couch model and the SBRT board was used as before. Dose calculation algorithm, CGS, convergence mode, and PO settings were identical to TrueBeam plans. However, jaw tracking was not available due to the jawless design of Halcyon RDS. All Halcyon plans were normalized to achieve identical combined target coverage to clinical TrueBeam plans providing similar target maximum dose.

### 2.3 Plan evaluation

Both TrueBeam and Halcyon plans were evaluated using NRG‐BR001 and RTOG‐0813 SBRT protocol requirements for target coverage and dose to OAR. For each lesion, target conformity index (CI) and Paddick conformation number (PCN)^33^ were determined using the ratio of the prescription isodose volume to the PTV and the ratio of the target volume covered by the prescription isodose squared to the product of the target volume and prescription isodose volume, respectively. The heterogeneity index (HI), used to evaluate plan hot spots, is the ratio of target maximum dose (*D*_max_) to the prescribed dose and the gradient index (GI) is the ratio of the 50% prescription isodose volume to the target volume. Additionally, intermediate‐dose fall‐off was assessed using maximum dose to any point 2 cm away from the target margin (*D*_2cm_) and recorded as percent of the prescription dose. As per RTOG requirements, percentage of normal lung receiving V20Gy was assessed along with the additional metrics of V5Gy, V10Gy, and mean lung dose (MLD). In addition to normal lung doses, maximal and volumetric dose to spinal cord, heart, esophagus, trachea/bronchial tree, ribs, and skin were evaluated per RTOG‐0813 and NRG‐BR001 guidelines. All dosimetric parameters where then compared between clinical TrueBeam plans and the fully reoptimized Halcyon plans.

### 2.4 Treatment delivery efficiency and accuracy

For each plan, delivery efficiency was assessed by evaluating the total number of MU per fraction and the beam modulation factor (MF) defined as the ratio of total number of MU to the fractional prescription dose in cGy. The actual beam‐on time was determined by dividing the total MU by the dose rate of 1400 MU/min for TrueBeam plans and 800 MU/min for Halcyon plans as observed on each plan. For a total of 50 Gy in five fractions prescription, each plan achieved the corresponding maximal dose rates for each control point for both machines. Total treatment time for TrueBeam plans incorporated: patient set up, 1‐min pre‐treatment CBCT, manual image matching, shift application, and beam‐on time; manual couch rotations during treatment for non‐coplanar arcs were not included in this estimate. For Halcyon RDS, total treatment time included: patient “one‐step set up”, 15‐s pre‐treatment kV‐iCBCT, automatic image matching and shift application, and beam‐on time. Delivery accuracy was evaluated by performing pre‐treatment portal dosimetry (PD) quality assurance (QA) measurements on the Linac used for each plan with a gamma evaluation criteria of 2%/2 mm and low‐dose threshold set to 5%.^34^, ^35^ The electronic portal imaging device (EPID, aS1200 flat panel detector; Varian Medical Systems) mounted on each machine includes a detector area of 40 cm × 40 cm providing a resolution of 0.34 mm was used for PD QA measurements. Additionally, overall treatment delivery time was estimated by incorporating the patient set up, CBCT imaging and registration, and patient set up verification times for each delivery platform as described above.

### 2.5 Data analysis

To assess the normality of each parameter, the Shapiro test followed by an evaluation of skewness and kurtosis was conducted. Comparison of dosimetric parameters and metrics was performed using the Wilcoxon rank test (nonparametric) or paired samples *t*‐test (parametric) and a significance level of *p*‐value of <0.05 in SPSS 27 data analysis software (IBM, New York, NY USA).

## 3 RESULTS

### 3.1 Target coverage and intermediate‐dose spillage

Plan quality and target metrics are displayed in Table 2 for both TrueBeam and Halcyon plans, each demonstrating compliance with NRG‐BR001 protocol requirements. Both plans produced statistically insignificant differences in target coverage, GTV doses (minimum, maximum, mean), CI, and PCN demonstrating similar target coverage can be achieved. Even with Halcyon's coplanar geometry, target dose, HI, GI, and *D*_2cm_ show slight but statistically insignificant improvements when compared to clinical TrueBeam plans.

**TABLE 2:** Analysis of the target metrics for all 16 lung stereotactic body radiation therapy (SBRT) patients treated with single‐isocenter/multiple‐lesions volumetric‐modulated arc therapy (VMAT) plans on TrueBeam compared to Halcyon

Target	Parameter	TrueBeam VMAT	Halcyon VMAT	*p*‐Value
PTV (*n* = 32)	% Volume covered by Rx dose (%)	96.6 ± 1.5 (95.1−99.6)	96.4 ± 1.3 (95.2–98.8)	0.476
	CI	1.06 ± 0.1 (0.89−1.29)	1.04 ± 0.09 (0.93−1.21)	0.054
	PCN	0.89 ± 0.06 (0.76−0.96)	0.91 ± 0.04 (0.77−0.96)	0.649
	HI	1.24 ± 0.05 (1.15−1.27)	1.22 ± 0.05 (1.14−1.31)	0.091
	GI	5.25 ± 1.06 (3.60−7.64)	5.13 ± 1.13 (3.69−8.12)	0.136
	*D*_2cm_ (%)	54.8 ± 5.9 (47.6−68.9)	54.1 ± 4.3 (47.2−65.8)	0.869
GTV (*n* = 32)	Minimum dose (Gy)	53.8 ± 2.9 (46.5−58.4)	53.5 ± 2.1 (50.4−58.0)	0.698
	Maximum dose (Gy)	61.1 ± 1.4 (57.5−63.5)	60.3 ± 2.2 (57.0−65.5)	0.114
	Mean dose (Gy)	57.1 ± 1.3 (54.9−60.8)	57.4 ± 1.8 (54.2−60.5)	0.122

*Note*: Mean ± SD (range) and *p*‐values were reported.

Abbreviations: CI, conformity index; GI, gradient index; GTV, gross tumor volume; HI, heterogeneity index; PCN, Paddick conformation number; PTV, planning target volume; SD, standard deviation.

### 3.2 Dose to normal lung

The dose to normal lung was evaluated using V20Gy as specified by the RTOG‐0813 protocol along with V5Gy, V10Gy, and MLD. The detail results for both plans are shown in Table 3. On average, Halcyon plans demonstrated statistically insignificant decreases in all normal lung metrics compared to clinical TrueBeam plans, for example, with V10Gy showing the largest reduction of 1.1%.

**TABLE 3:** Normal lung dose statistics between single‐isocenter TrueBeam volumetric‐modulated arc therapy (VMAT) and Halcyon VMAT plans for all 16 lung stereotactic body radiation therapy (SBRT) patients with two tumors

Plan type	V20Gy (%)	V10Gy (%)	V5Gy (%)	MLD (Gy)
TrueBeam VMAT	8.8 ± 4.8 (2.7−14.9)	23.0 ± 11.7 (7.8−43.4)	34.3 ± 14.6 (11.6−56.3)	6.5 ± 2.8 (2.4−10.9)
Halcyon VMAT	8.5 ± 4.7 (2.5−15.2)	21.9 ± 11.1 (8.2−37.6)	34.5 ± 14.7 (12.4−56.6)	6.4 ± 2.6 (2.4−9.8)
*p*‐Value	0.387	0.145	0.942	0.466

*Note*: Mean ± SD (range) and *p*‐values were reported.

Abbreviations: MLD, mean lung dose; SD, standard deviation.

### 3.3 Dose to other OARs

**FIGURE 1:**
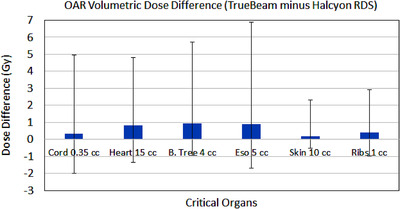
Organs at risk (OAR) volumetric dose differences (TrueBeam minus Halcyon) for all 16 synchronous volumetric‐modulated arc therapy (VMAT) lung stereotactic body radiation therapy (SBRT) patients. Mean difference is the blue box plot with upper and lower boundaries are the maximum and minimum dose differences. On average, Halcyon plans yielded dose reductions to all critical organs including 15 cc of heart, 4 cc of bronchial tree, 5 cc of esophagus, and a statistically significant decrease in dose to 1 cc of ribs and 5 cc of esophagus. Dose to 10 cc of skin and 0.35 cc of spinal cord show minimal change in Halcyon plans due to coplanar geometry

As per RTOG‐0813/BR001 protocol requirements, maximal and volumetric dose to OAR (cord, heart, esophagus, trachea/bronchus, skin, ribs) were recorded for both TrueBeam and Halcyon plans. Paired volumetric differences for all OAR with respect to TrueBeam plans are presented graphically in Figure 1 and tabulated results for volumetric and maximal dose are shown in Table 4. Positive values indicate that Halcyon plans decreased OAR dose when compared to the clinical TrueBeam plans. Both plans met all protocol compliance criteria for OAR sparing and were clinically acceptable. Most comparisons yielded statistically insignificant differences with the exception of an average dose reduction to 1 cc and maximal dose to ribs of 0.43 Gy (*p* = 0.033) and 1.50 Gy (*p* = 0.041), respectively. The maximal dose reduction to the heart of 3.36 Gy (*p* = 0.025) and dose to 5 cc of esophagus was 0.9 Gy (*p* = 0.043) in Halcyon plans, respectively. It is also important to note that although not statistically significant, on average, Halcyon plans provided additional dose reductions of 0.81 Gy to the heart (15 cc), 1.3 Gy maximal dose to the esophagus, and 0.95 Gy (4 cc) and 0.51 Gy (maximal dose) to the proximal bronchial tree indicating clinically significant dose reductions.

**TABLE 4:** Evaluation of dose to organs at risk (OAR) for all 16 two‐lesion lung stereotactic body radiation therapy (SBRT) patients for both plans

Dose to OAR	Parameters	Difference = TrueBeam minus Halcyon plans	*p*‐Value
Spinal cord (Gy)	*D*_max_	0.13 ± 2.35 (‐2.21 to 5.60)	0.910
	*D*_0.35cc_	0.33 ± 1.98 (‐2.34 to 4.64)	0.956
Heart/pericardium (Gy)	*D*_max_	3.36 ± 4.49 (‐1.72 to 14.08)	**0.026**
	*D*_15cc_	0.81 ± 1.71 (‐2.15 to 4.01)	0.063
Esophagus (Gy)	*D*_max_	1.30 ± 3.43 (‐3.39 to 8.01)	0.275
	*D*_5cc_	0.89 ± 2.22 (‐2.56 to 6.14)	**0.043**
Trachea/bronchus (Gy)	*D*_max_	0.51 ± 3.91 (‐4.50 to 8.15)	0.681
	*D*_4cc_	0.95 ± 2.14 (‐1.89 to 4.75)	0.136
Skin (Gy)	*D*_max_	0.18 ± 2.06 (‐3.01 to 3.23)	0.733
	*D*_10cc_	0.17 ± 0.88 (‐0.69 to 2.14)	0.286
Ribs (Gy)	*D*_max_	1.50 ± 2.61 (‐1.57 to 7.07)	**0.041**
	*D*_1cc_	0.43 ± 1.23 (‐1.39 to 2.59)	**0.033**

*Note*: Mean ± SD (range) was reported. Statistically significant *p*‐values are highlighted in bold.

Abbreviation: SD, standard deviation.

### 3.4 Treatment delivery efficiency and accuracy

Analysis of treatment delivery efficiency was performed for both TrueBeam and Halcyon plans by comparing the mean total number of MU and its associated metrics of beam MF, beam‐on time, and overall treatment time (see Table 5). When compared to clinical TrueBeam plans, Halcyon plans provided increased total MU on average by 480 MU (*p* = 0.041) thus resulting in a corresponding increase in MLC MF of 0.48. On average, beam‐on time also increased in Halcyon plans by 2.78 min (*p* < 0.001) reflecting the effect of lower maximal dose rate of Halcyon (800 MU/min) when compared to TrueBeam (1400 MU/min). However, mean overall treatment time did decrease in Halcyon plans by 2.22 min (range, 1.38–3.41 min) due to several factors as mentioned above. When compared to TrueBeam's 1‐min CBCT scan times and manual image matching procedure, Halcyon RDS offers a gantry rotation speed up to four times faster than TrueBeam and a more streamlined fully automated “one‐step patient set up” with a high‐quality 15‐s kV‐iCBCT scanning capability and auto‐image matching feature.

**TABLE 5:** Comparison of average values (and range) of treatment delivery parameters between clinical TrueBeam volumetric‐modulated arc therapy (VMAT) and Halcyon plans for all 16 lung stereotactic body radiation therapy (SBRT) patients with two lesions

Beam delivery parameters	TrueBeam VMAT plans	Halcyon VMAT plans	*p*‐Value
Total MU/fraction	4052 ± 702 (2770−5439)	4532 ± 890 (2852−6007)	**0.041**
Modulation factor	4.05 ± 0.71 (2.77−5.44)	4.53 ± 0.89 (2.85−6.01)	**0.043**
Beam‐on time (min)	2.89 ± 0.50 (1.98−3.89)	5.67 ± 1.11 (3.57−7.51)	**<0.001**
Treatment time (min)	12.89 ± 0.51 (11.98−13.89)	10.67 ± 1.12 (8.57−12.51)	**<0.001**
Pre‐treatment PD QA pass rates (%) for (2%/2 mm)	98.9 ± 0.85 (98.1−100)	98.45 ± 0.99 (97.9−100)	0.064

*Note*: Mean ± SD (range) was reported. Statistically significant *p*‐values are highlighted in bold.

Abbreviations: MU, monitor units; PD, portal dosimetry; QA, quality assurance; SD, standard deviation.

As mentioned previously, treatment delivery accuracy of both Halcyon and clinical TrueBeam plans was assessed via treatment plan delivery in patient‐specific QA measurement mode on both Linacs using the respective on‐board EPID imager followed by evaluation of gamma pass rates by PD. Mean dose delivery accuracy of TrueBeam clinical plans and the corresponding Halcyon plans was 98.9 ± 0.85% (range 98.1%–100%) and 98.5 ± 0.99% (range 97.9%–100%), respectively, demonstrating a statistically insignificant decrease in the QA pass rates for Halcyon plans even with slightly higher beam modulation. Due to the higher pixel resolution of the aS1200 EPID detector (0.34 mm) and its limited ability to identify the small dosimetric differences for 3%/3 mm gamma clinical criteria, ≥95% pass rates with 2%/2 mm gamma criteria was used for analyzing the data.

### 3.5 Bilateral lesions example case

**FIGURE 2:**
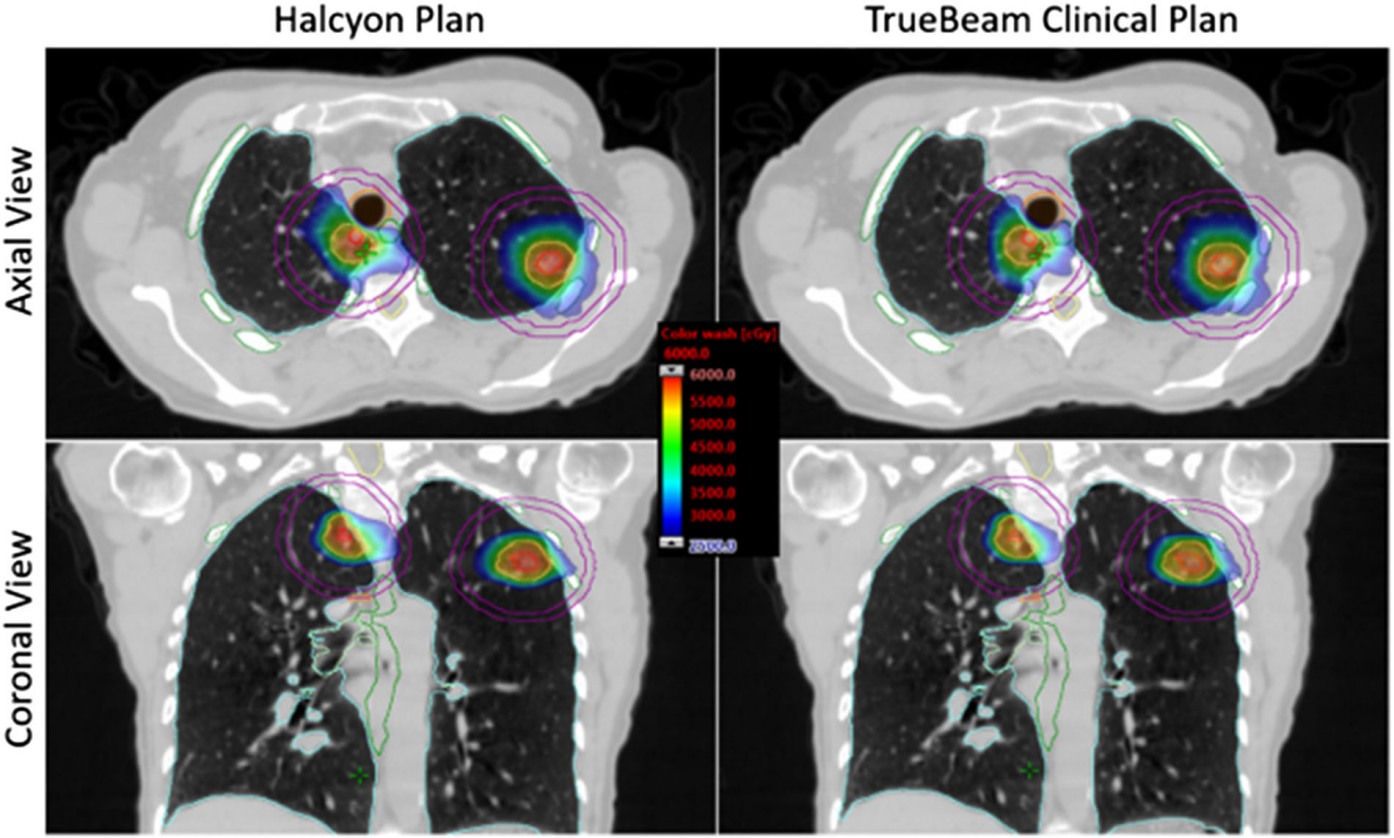
Axial and coronal plane of an example patient's clinical plan (right panel) and Halcyon plan (left panel) using a single‐isocenter placed between the lesions. Shown is gross tumor volumes (GTVs) (red), planning target volumes (PTVs) (pink), ribs (green), cord (yellow), esophagus (green), trachea (orange), bronchus (light green), and normal lung (cyan). Isodose color wash (25–60 Gy) and *D*_2cm_ ring (purple) to each tumor shows similar target conformity and intermediate‐dose spillage to non‐coplanar TrueBeam at minimal cost to plan complexity

**FIGURE 3:**
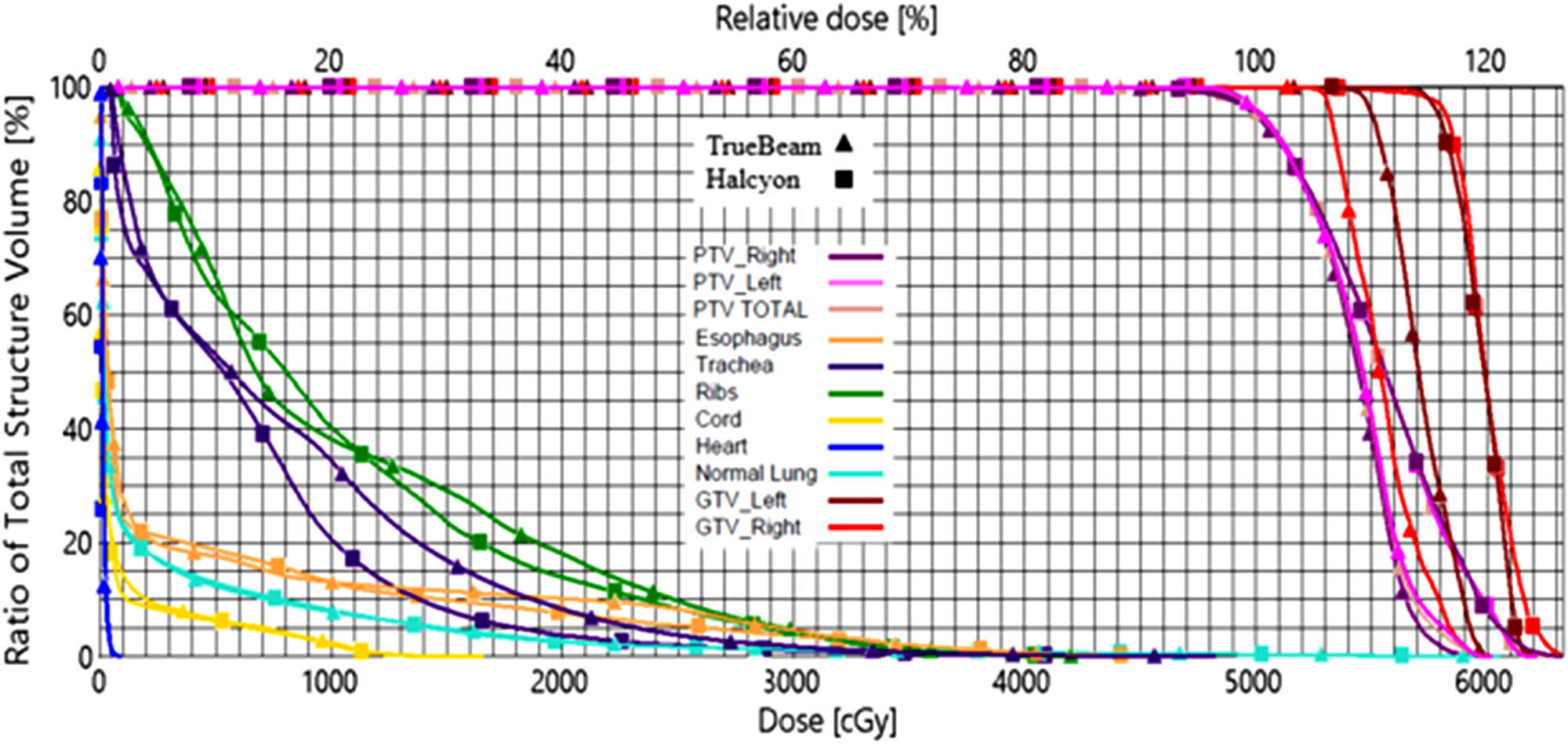
Dose volume histogram for the example patient shown in Figure 2. The patient had bilateral lung lesions that were treated synchronously. The triangles represent the clinical TrueBeam plan and squares represent Halcyon plan. Structures shown are gross tumor volumes (GTVs) (red and dark red), planning target volumes (PTVs) (pink and magenta), ribs (green), heart (blue), cord (yellow), esophagus (orange), trachea (purple), and normal lung (cyan). For this patient, Halcyon plan provided substantial GTV dose escalation to both lesions while maintaining comparable intermediate/high‐dose spillage and similar or better organs at risk (OAR) sparing

An example patient from the cohort representing the typical findings of the study is illustrated in Figures 2 and 3. This patient had bilateral lung lesions located in the right upper lobe (RUL) and left upper lobe (LUL). The combined PTV was 20.5 cc (RUL: 7.3 cc and LUL: 13.2 cc) with an average distance from isocenter to the center of the lesion of 5.3 cm. The TrueBeam clinical plan utilized three partial arcs, two coplanar, and one non‐coplanar (10° couch rotation), with an arc length of 150° and three different collimator rotations. For the Halcyon plan, three coplanar partial arcs were used with an arc length of 270° and three different collimator rotations. For this patient, TrueBeam yielded a combined CI, HI, GI, *D*_2cm_, and V20Gy of 1.03, 1.20, 5.6, 50.6%, and 2.7% versus 1.05, 1.24, 5.6, 55.4%, and 2.5% for the Halcyon plan. All maximal and volumetric doses to OAR were within RTOG‐0813/BR001 compliance criteria. Total MU per fraction, MF, beam‐on time, and treatment time for TrueBeam was 3796, 3.8, 2.71 min, and 12.71 min compared to Halcyon's 4608, 4.61, 5.75 min, and 10.76 min, respectively. Figure 2 shows the dose distributions of both plans in axial and coronal planes for the example patient. As is evident by the increase in *D*_2cm_, the dose color wash (Figure 2) shows that the coplanar geometry of Halcyon produces a slight elongation of the 50% isodose for the lesion in the RUL compared to the non‐coplanar TrueBeam plan. However, as shown by the cumulative dose volume histogram in Figure 3, the Halcyon plan is still able to provide clinically significant dose escalation to both GTVs while providing similar or better OAR sparing.

## 4 DISCUSSION

We evaluated the plan quality, delivery efficiency, and accuracy of VMAT plans via a novel Halcyon RDS in the treatment of single‐isocenter/two‐lesion lung SBRT following RTOG‐0813/NRG‐BR001 protocol requirements and comparison with clinically delivered non‐coplanar/coplanar TrueBeam plans. Comparison with clinical TrueBeam plans provided a reference for achievable plan quality at our clinic for the highest quality of patient treatment. Our findings suggest that for select lung cancer patients, Halcyon RDS is capable of delivering highly conformal single‐isocenter/two‐lesion lung SBRT treatments that meet all the RTOG‐0813/NRG‐BR001 compliance criteria and are dosimetrically similar to our SBRT‐dedicated TrueBeam Linac in terms of plan quality and deliverability. Specifically, Halcyon produced no statistical or clinically significant differences in target metrics at no additional cost to OAR dose sparing. While Halcyon plans required slightly more MU (480, on average), pre‐treatment QA suggest similar plan deliverability and treatment accuracy can be achieved. We estimated Halcyon reduced overall treatment time by 2.22 min on average despite the MU increase because enhanced built in fully automated patient set up capabilities permit time saving. As mentioned earlier, this is due to therapists not having to enter the room to manually apply the shifts or couch rotations as is the case for non‐coplanar VMAT arcs in TrueBeam plans, as well as avoiding patient collision issue on Halcyon RDS.

In the past years, the efficacy of the single‐isocenter approach to treating multiple lung lesions has been examined by several studies.^15^, ^36^, ^37^, ^38^ In one of the first studies to evaluate this approach in the lung, Zhang et al.^36^ compared intensity modulated radiation therapy (IMRT) treatments consisting of coplanar and non‐coplanar field geometries with helical tomotherapy in the treatment of multiple lung lesions using a single‐isocenter plan. Although the results showed improved target coverage and increased dose sparing of adjacent OAR, average treatment time was well over an hour with increased low‐dose volumes that could present toxicity concerns in normal lung tissue. More recently, Quan et al.^37^ reported a study that contained 11 patients with two or more lesions that received 30–54 Gy in three to five fractions using the single‐isocenter technique. This study produced high‐quality clinical VMAT plans and substantial reductions in treatment time when compared to a traditional multi‐isocentric approach. Furthermore, Sanford et al.^15^ implemented a single‐isocentric VMAT planning technique clinically, using TrueBeam Linac and a 6MV‐FFF beam to treat eight patients with two peripherally located lung lesions with prescription doses of 50–54 Gy in three to five fractions. They reported similar dosimetric plan quality to two‐isocenter plans and significant reductions in treatment time, however, they did observe small increases in normal lung V5Gy, V10Gy, and MLD as the distance between lesions increased. However, in a clinical follow up results to that study, Pokhrel et al.^38^ reported 100% local control rates and no treatment‐related acute toxicity (mean, 9 months) thus mitigating the concerns of these slight dose increases.

Halcyon may improve single‐isocenter/multiple‐lesion lung SBRT treatment with respect to previous studies conducted with helical tomotherapy.^36^, ^39^ In a retrospective planning study conducted by Deng et al.^40^ that evaluates the performance of Halcyon RDS in the treatment of multiple brain lesions with a single‐isocenter plan, the study compared dynamic conformal arc (DCA) and TrueBeam HDMLC (2.5 mm leaf) coplanar and non‐coplanar plans with the Halcyon 2.0 VMAT plans. The Halcyon plan yielded similar conformity, but inferior GI compared to clinical DCA coplanar and non‐coplanar plans for lesions larger than 1.0 cm in diameter. They also observed reduced low‐dose spillage for Halcyon plans compared to TrueBeam coplanar and non‐coplanar plans. In contrast to our study, it is important to note that dosimetric characteristics in the brain will not necessarily translate to the heterogeneities present in the lung tumors. In some cases, our results do show slightly inferior *D*_2cm_ and GI due to the coplanar limitation of Halcyon RDS. However, reduced MLC transmission has been shown to be a dominant factor in increased OAR sparing in highly modulated plans as reported by Li et al.,^29^ that was similar to our study. Therefore, in addition to faster MLC speed and given Halcyon's ultra‐low MLC leakage and transmission dose (∼0.4%) compared to the higher leakage/transmission of TrueBeam 6MV‐FFF beam (1.5%), it is reasonable to conclude that for some treatment geometries, Halcyon RDS should be capable of similar or better normal tissue sparing despite the increased degree of modulation observed in this study.

Although this study shows promise for future treatments of single‐isocenter/two‐lesion VMAT lung SBRT on Halcyon RDS, it does have some limitations. Most importantly, Halcyon currently is unable to apply rotational corrections to the treatment couch position, limiting patient set up verification to only three translational corrections. Our TrueBeam however, is equipped with a perfect‐pitch couch allowing both rotational and translational corrections to patient set up in treatments of synchronous lung lesions. The dosimetric impact of this limitation is not currently known for Halcyon RDS and is currently under investigation. Another limitation observed in this study is that Halcyon's maximum achievable dose rate of 800 MU/min is substantially lower than TrueBeam's maximum dose rate of up to 1400 MU/min when using 6MV‐FFF beam. Although in this study we observed the maximum dose rate throughout all the control points in the TrueBeam treatment delivery, the slower gantry rotation speed can decrease the treatment time throughout the treatment whereas Halcyon is able to maintain delivery at the maximum dose rate and up to four times faster the gantry rotation speed. An upgrade of Halcyon's maximal achievable dose rate of up to 1000 MU/min could potentially result in beam‐on times much closer or even faster to that of current TrueBeam delivery and further reduce overall treatment times.

In summary, we have demonstrated that use of Halcyon RDS for VMAT to deliver synchronous SBRT treatment to two lung lesions using a single‐isocenter plan is not only feasible but can potentially be advantageous for select lung cancer patients who may not tolerate traditional longer SBRT treatment times due to back pain, distress and discomfort, or shortness of breath. Halcyon's enhanced MLC design along with ultra‐low leaf transmission/leakage and increased travel speeds result in similar target coverage, intermediate‐ and high‐dose spill, and similar or better dose to adjacent OAR. Moreover, Halcyon RDS requires minimal patient set up time when compared to TrueBeam and is capable of gantry rotation speeds of up to 4 revolutions/min resulting in a clinically significant reduction in overall treatment time, potentially improving patient comfort and clinic workflow. This benefit could potentially reduce intrafraction motion and the resulting dosimetric error, which has been shown to increase linearly with treatment time.^14^ Based on this research and our previous studies, we plan on continuing our efforts to define the capabilities and limitations of Halcyon RDS by quantifying the dosimetric impact due to the lack of rotational corrections as well as the detail effects of the dual‐layer MLC in the future investigations.

## 5 CONCLUSION

This study has demonstrated the efficacy of single‐isocenter/two‐lesion VMAT lung SBRT delivery on the Halcyon RDS for the select lung cancer patients. The results indicate that Halcyon can provide safe, effective, and accurate treatments that are dosimetrically equivalent or potentially preferable when compared to SBRT‐dedicated TrueBeam plans with minimal or no additional cost in plan complexity and dose to OAR. For clinics equipped only with Halcyon, this could provide an additional treatment capability for the patient cohort located in underserved areas and unable to travel to larger treatment centers due to physical or financial limitations. Conversely, busy treatment centers with Halcyon RDS, currently employing C‐arm Linacs for this technique, could provide an additional platform for lung SBRT treatment delivery. Additionally, for select lung cancer patients who may not tolerate longer treatment times due to back pain, shortness of breath, and discomfort, Halcyon's highly automated patient set up coupled with faster gantry speed offers a reduction in overall treatment time, potentially reducing dosimetric error due to intrafraction patient motion. However, for two‐lesion lung SBRT patients, further investigation should be conducted to determine the dosimetric impacts of Halcyon's inability to correct rotational patient set up error.

## AUTHOR CONTRIBUTIONS

Damodar Pokhrel and Aaron Webster conceptualized the project. Aaron Webster and Damodar Pokhrel generated two‐lesion lung SBRT plans on Halcyon RDS. Aaron Webster, Richard Mallory, Damodar Pokhrel, and Justin Visak collected and analyzed the data. Damodar Pokhrel, Mark E. Bernard, Ronald C. McGarry, and Mahesh Kudrimoti provided clinical expertise and supervision of the paper. Damodar Pokhrel and Aaron Webster drafted the preliminary version of the manuscript and all co‐authors revised and approved the final manuscript.

## CONFLICT OF INTEREST

None.

## Data Availability

Research data are not shared.

## References

[1] Siegel RL , Miller KD , Jemal A . Cancer statistics, 2020. CA Cancer J Clin. 2020;70(1):7‐30. 10.3322/caac.21590 31912902

[2] Navarria P , Ascolese AM , Mancosu P , et al. Volumetric modulated arc therapy with flattening filter free (FFF) beams for stereotactic body radiation therapy (SBRT) in patients with medically inoperable early stage non small cell lung cancer (NSCLC). Radiother Oncol. 2013;107(3):414‐418. 10.1016/j.radonc.2013.04.016 23725859

[3] Bradley J , Gaspar L , Capote R , et al. Radiation therapy oncology group 0813 seamless phase I/II study of stereotactic lung radiotherapy (SBRT) for early stage, centrally located, non‐small cell lung cancer (NSCLC) in medically inoperable patients. Lung. 2006:19.

[4] Timmerman R , Paulus R , Galvin J , et al. Stereotactic body radiation therapy for inoperable early stage lung cancer. J Am Med Assoc. 2010;303(11):1070‐1076. 10.1001/jama.2010.261 PMC2907644 20233825

[5] Cai B , Laugeman E , Mazur TR , et al. Characterization of a prototype rapid kilovoltage x‐ray image guidance system designed for a ring shape radiation therapy unit. Med Phys. 2019;46(3):1355‐1370. 10.1002/mp.13396 30675902 PMC8188470

[6] Simone CB , Wildt B , Haas AR , Pope G , Rengan R , Hahn SM . Stereotactic body radiation therapy for lung cancer. J Am Med Assoc. 2013;143(6):1784‐1790. 10.1378/chest.12-2580 23732589

[7] Rusthoven KE , Pugh TJ . Stereotactic body radiation therapy for inoperable lung cancer. J Am Med Assoc. 2010;303(23):2354‐2355. 10.1001/jama.2010.777 20551403

[8] Onishi H , Shirato H , Nagata Y , et al. Stereotactic body radiotherapy (SBRT) for operable stage I non‐small‐cell lung cancer: can SBRT be comparable to surgery? Int J Radiat Oncol Biol Phys. 2011;81(5):1352‐1358. 10.1016/j.ijrobp.2009.07.1751 20638194

[9] Rusthoven KE , Kavanagh BD , Burri SH , et al. Multi‐institutional phase I/II trial of stereotactic body radiation therapy for lung metastases. J Clin Oncol. 2009;27(10):1579‐1584. 10.1200/jco.2008.19.6386 19255320

[10] Okunieff P , Petersen AL , Philip A , et al. Stereotactic body radiation therapy (SBRT) for lung metastases. Acta Oncol. 2006;45:808‐817. 10.1080/02841860600908954 16982544

[11] Al‐Hallaq HA , Chmura S , Salama JK , et al. Rationale of technical requirements for NRG‐BR001: the first NCI‐sponsored trial of SBRT for the treatment of multiple metastases. Pr Radiat Oncol Oncol. 2016;6(6):e291‐e298. 10.1016/j.prro.2016.05.004 PMC5099083 27345129

[12] Videtic GM , Paulus R , Singh AK , et al. Long‐term follow‐up on NRG oncology RTOG 0915 (NCCTG N0927): a randomized phase 2 study comparing 2 stereotactic body radiation therapy schedules for medically inoperable patients with stage I peripheral non‐small cell lung cancer. Clin Invest. 2019;103:1077‐1087. 10.1016/j.ijrobp.2018.11.051 PMC6454873 30513377

[13] Benedict SH , Yenice KM , Followill D , et al. Stereotactic body radiation therapy: the report of AAPM Task Group 101. Med Phys. 2010;37(8):4078‐4101. 10.1118/1.3438081 20879569

[14] Hoogeman MS , Nuyttens JJ , Levendag PC , Heijmen BJM . Time dependence of intrafraction patient motion assessed by repeat stereoscopic imaging. Int J Radiat Oncol Biol Phys. 2008;70(2):609‐618. 10.1016/j.ijrobp.2007.08.066 17996389

[15] Sanford L , Molloy J , Kumar S , Randall M , McGarry R , Pokhrel D . Evaluation of plan quality and treatment efficiency for single‐isocenter/two‐lesion lung stereotactic body radiation therapy. J Appl Clin Med Phys. 2019;20(1):118‐127. 10.1002/acm2.12500 PMC6333146 30548205

[16] Merrow CE , Wang IZ , Podgorsak MB . A dosimetric evaluation of VMAT for the treatment of non‐small cell lung cancer. J Appl Clin Med Phys. 2013;14(1):228‐238. 10.1120/jacmp.v14i1.4110 PMC5714051 23318374

[17] Critchfield LCS , Bernard ME , Randall ME , McGarry RC , Pokhrel D . Risk of target coverage loss for stereotactic body radiotherapy treatment of synchronous lung lesions via single‐isocenter volumetric modulated arc therapy. J Appl Clin Med Phys. 2021;22(1):251‐260. 10.1002/acm2.13145 PMC7856510 33342042

[18] Ong CL , Verbakel WFAR , Cuijpers JP , Slotman BJ , Lagerwaard FJ , Senan S . Stereotactic radiotherapy for peripheral lung tumors: a comparison of volumetric modulated arc therapy with 3 other delivery techniques. Radiother Oncol. 2010;97(3):437‐442. 10.1016/j.radonc.2010.09.027 21074878

[19] Nagai A , Shibamoto Y , Yoshida M , Inoda K , Kikuchi Y . Safety and efficacy of intensity‐modulated stereotactic body radiotherapy using helical tomotherapy for lung cancer and lung metastasis. Biomed Res Int. 2014;2014:473173. 10.1155/2014/473173 PMC4065754 24995299

[20] Pokhrel D , Halfman M , Sanford L . FFF‐VMAT for SBRT of lung lesions: improves dose coverage at tumor‐lung interface compared to flattened beams. J Appl Clin Med Phys. 2020;21(1):26‐35. 10.1002/acm2.12764 PMC6964748 31859456

[21] Varian Medical Systems. *Halcyon Physics 1.0*. 2017:1‐183.

[22] Lim TY , Dragojević I , Hoffman D , Flores‐Martinez E , Kim GY . Characterization of the Halcyon TM multileaf collimator system. J Appl Clin Med Phys. 2019;20(4):106‐114. 10.1002/acm2.12568 30889312 PMC6448159

[23] Tamura M , Matsumoto K , Otsuka M , Monzen H . Plan complexity quantification of dual‐layer multi‐leaf collimator for volumetric modulated arc therapy with Halcyon linac. Phys Eng Sci Med. 2020;43(3):947‐957. 10.1007/s13246-020-00891-2 32648112

[24] Jarema T , Aland T . Using the iterative kV CBCT reconstruction on the Varian Halcyon linear accelerator for radiation therapy planning for pelvis patients. Phys Med. 2019;68:112‐116. 10.1016/j.ejmp.2019.11.015 31783220

[25] Li T , Scheuermann R , Lin A , et al. Impact of multi‐leaf collimator parameters on head and neck plan quality and delivery: a comparison between Halcyon™ and Truebeam® treatment delivery systems. Cureus. 2018;10(11):1‐9. 10.7759/cureus.3648 PMC6351111 30723647

[26] Knutson NC , Kennedy WR , Reynoso FJ , et al. Intracranial stereotactic radiation therapy with a jawless ring gantry linear accelerator equipped with new dual layer multileaf collimator. Adv Radiat Oncol. 2020;5(3):482‐489. 10.1016/j.adro.2020.01.003 32529144 PMC7276691

[27] Petroccia HM , Malajovich I , Barsky AR , et al. Spine SBRT with Halcyon™: plan quality, modulation complexity, delivery accuracy, and speed. Front Oncol. 2019;9:1‐8. 10.3389/fonc.2019.00319 31106151 PMC6498946

[28] Pokhrel D , Tackett T , Stephen J , et al. Prostate SBRT using O‐Ring Halcyon Linac — plan quality, delivery efficiency, and accuracy. J Appl Clin Med Phys. 2021;22(1):68‐75. 10.1002/acm2.13105 33340388 PMC7856496

[29] Li T , Irmen P , Liu H , et al. Dosimetric performance and planning/delivery efficiency of a dual‐layer stacked and staggered MLC on treating multiple small targets: a planning study based on single‐isocenter multi‐target stereotactic radiosurgery (SRS) to brain metastases. Front Oncol. 2019;9:7. 10.3389/fonc.2019.00007 PMC6349708 30723702

[30] Pokhrel D , Visak J , Critchfield LC , et al. Clinical validation of ring‐mounted halcyon linac for lung SBRT: comparison to SBRT‐dedicated C‐arm linac treatments. J Appl Clin Med Phys. 2021;22(1):261‐270. 10.1002/acm2.13146 PMC7856490 33342070

[31] Vassiliev ON , Wareing TA , McGhee J , Failla G , Salehpour MR , Mourtada F . Validation of a new grid‐based Boltzmann equation solver for dose calculation in radiotherapy with photon beams. Phys Med Biol. 2010;55(3):581‐598. 10.1088/0031-9155/55/3/002 20057008

[32] Kroon PS , Hol S , Essers M . Dosimetric accuracy and clinical quality of Acuros XB and AAA dose calculation algorithm for stereotactic and conventional lung volumetric modulated arc therapy plans. Radiat Oncol. 2013;8(1): 1. 10.1186/1748-717X-8-149 PMC3723919 23800024

[33] Paddick I . A simple scoring ratio to index the conformity of radiosurgical treatment plans. Technical note. J Neurosurg. 2000;93(suppl 3):219‐222. 10.3171/jns.2000.93.supplement_3.0219 11143252

[34] Laugeman E , Heermann A , Hilliard J , et al. Comprehensive validation of halcyon 2.0 plans and the implementation of patient specific QA with multiple detector platforms. J Appl Clin Med Phys. 2020;21(7):39‐48. 10.1002/acm2.12881 PMC7386180 32368862

[35] Miri N , Keller P , Zwan BJ , Greer P . EPID‐based dosimetry to verify IMRT planar dose distribution for the aS1200 EPID and FFF beams. J Appl Clin Med Phys. 2016;17(6):292‐304. 10.1120/jacmp.v17i6.6336 27929502 PMC5690494

[36] Zhang Y , Chen Y , Qiu J , Yang J . Dosimetric comparisons of lung SBRT with multiple metastases by two advanced planning systems. Int J Med Phys, Clin Eng Radiat Oncol. 2014;03(04):252‐261. 10.4236/ijmpcero.2014.34032

[37] Quan K , Xu KM , Lalonde R , et al. Treatment plan technique and quality for single‐isocenter stereotactic ablative radiotherapy of multiple lung lesions with volumetric‐modulated arc therapy or intensity‐modulated radiosurgery. Front Oncol. 2015;5:1‐9. 10.3389/fonc.2015.00213 26500888 PMC4594030

[38] Pokhrel D , Sanford L , Larkin S , et al. On the use of single‐isocenter VMAT plans for SBRT treatment of synchronous multiple lung lesions: plan quality, treatment efficiency, and early clinical outcomes. J Appl Clin Med Phys. 2020;21(8):160‐167. 10.1002/acm2.12938 32432405

[39] Sterzing F , Welzel T , Sroka‐Perez G , Schubert K , Debus J , Herfarth KK , et al. Reirradiation of multiple brain metastases with helical tomotherapy. A multifocal simultaneous integrated boost for eight or more lesions. Strahlenther Onkol. 2009;185(2):89‐93. 10.1007/s00066-009-1971-2 19240994

[40] Deng J , Zhang Y , Zhou L , et al. Dosimetric performance and planning/delivery efficiency of a dual‐layer stacked and staggered MLC on treating multiple small targets: a planning study based on single‐isocenter multi‐target stereotactic radiosurgery (SRS) to brain metastases. Front Oncol. 2019;1: 7. 10.3389/fonc.2019.00007 PMC6349708 30723702

